# Editorial: Case reports in neonatology 2022

**DOI:** 10.3389/fped.2023.1200414

**Published:** 2023-04-20

**Authors:** Sascha Meyer, Michael Wagner, Merih Cetinkaya

**Affiliations:** ^1^ Department of General Pediatrics and Neonatology, Franz-Lust Klinik für Kinder und Jugendliche, Karlsruhe, Germany; ^2^ Department of Neonatology, Medical University of Vienna, Vienna, Austria; ^3^ Department of Neonatology, University of Health Sciences, Istanbul, Türkiye

**Keywords:** neonatology, case reports, evidence-based medicine, randomized controlled trial, neonatal intensive care

**Editorial on the Research Topic** Case reports in neonatology 2022

This Research Topic publishes high-quality case reports and case series in the field of neonatology, ranging from lethal poisoning, adverse drug reactions, congenital pathologies, neonatal metabolic disease, survival and care practices of periviable births to malignancies in newborns (Baptiste et al. Cheng et al. Gaertner et al. Boddu et al. Chen et al. Yan et al.). Case reports provide important insights into the differential diagnosis, decision making, and clinical management of unusual cases. They provide a valuable tool for (a) recognizing new or rare diseases, (b) evaluating therapeutic effects, adverse events, and costs of interventions; as well as (c) improving problem-based medical education in a real-world clinical setting, Conversely, randomized clinical trials generate evidence for the efficacy of interventions in a controlled setting. Undoubtedly, both types of reports are necessary and complementary in modern medicine. Of note, case reports today make up a substantial proportion of articles published in medical journals, and they continue to further our understanding of a great variety of diseases. Consequently, case reports are an important contributor to and driver of medical progress. The CARE guidelines help increase the completeness, accuracy, and transparency of published case reports (1).

The importance of having access to case reports in our continuously changing modern world is key in the provision of best clinical care. In the immortal words of the famous Canadian physician, first Professor of Medicine and founder of the Medical Service at Johns Hopkins Hospital, Sir William Osler (July 12, 1849 to December 29, 1919): “Always note and record the unusual … Publish it. Save it on a permanent record as a short, concise note. Such communications are always of value” (2).

No doubt, the value of case reports may be underestimated compared to other publications that are more detailed and supported by evidence (eg, randomized controlled clinical trials). Nevertheless, this experience-based informative method has nowadays been transformed into an accepted academic form of communication and publication, thus contributing to the rapid dissemination of medical knowledge to a wide medical professional audience, including neonatologists.

Therefore, we hope that this Special Collection is both informative and stresses the importance and role of case reports in the field of neonatology, and will also translate into improved neonatal care practices around the world.
